# Physiological Changes of Bamboo (*Fargesia yunnanensis*) Shoots During Storage and the Related Cold Storage Mechanisms

**DOI:** 10.3389/fpls.2021.731977

**Published:** 2021-09-03

**Authors:** Lixia Yu, Jialong Pei, Yihe Zhao, Shuguang Wang

**Affiliations:** ^1^Key Laboratory for Sympodial Bamboo Research, Faculty of Life Sciences, Southwest Forestry University, Kunming, China; ^2^Institute of Forest Products Industry, Yunnan Academy of Forestry and Grassland, Kunming, China

**Keywords:** *Fargesia yunnanensis* shoots, postharvest storage, degradation, browning, fibrosis, lignification

## Abstract

The mechanisms for physiological senescence in bamboo shoots after harvest remain unclear. This study investigated physiological changes in *Fargesia yunnanensis* shoots during storage at different temperatures. The relationship between morphological and physiological changes in bamboo shoots during storage was also analyzed. The results show that cold storage can reduce weight loss, browning, respiration rates, and sugar degradation in bamboo shoots; decrease related enzymatic activities; and inhibit the increase in lignin and cellulose content. The quality of bamboo shoots declines more during the first 3d after harvesting than it does during subsequent periods. The increase in the degree of lignification and fibrosis is the main reason for senescence and for the decline in quality of bamboo shoots after harvest. The bamboo shoots under RT conditions began browning during the third 3d of storage, with a browning rate of 688gkg^−1^ even in the upper parts; the increase in shoot browning degrees significantly decreased the quality. Low temperatures had better inhibitory effects on browning than they did on lignification and fibrosis. Nonstructural carbohydrates in bamboo shoots are degraded and flow into sheath and shoot respiration, phenols, and shoot fibrosis and lignification at room temperature, but only flow into sheath respiration, shoot fibrosis, and lignification at cold temperature. Soluble protein and free amino acids are primarily distributed into shoot and sheath respiration and into phenols at room temperature, but that process is well inhibited at cold temperature. Bamboo shoots, once removed from cold storage, should be consumed rapidly because enzyme activity recovers quickly. This research provides new theoretical information on the preservation of bamboo shoots.

## Highlights

- Respiration results in the senescence and deterioration of shoots
- Bamboo shoots can stay fresh for 6d in cold storage
- Browning and lignification compete in phenol consumption

## Introduction

There are about 1,400 bamboo species in the world, and most produce edible shoots (Yeasmin et al., 2015). Bamboo shoots are an important vegetable in Asian countries with high nutritive values (Singh and Das, 2011; Liu et al., 2016) and are usually considered as a highly desirable food because of their low cholesterol and fat content, and more carbohydrates, antioxidant and antithyroidal properties (Kalita and Dutta, 2012). Bamboo shoots possess high protein, essential amino acids, carbohydrate, dietary fiber, and a potent antioxidant (Nirmala et al., 2007; Singhal et al., 2013). Bamboo shoots also have huge economical values. The worldwide economic potential for bamboo is estimated at US$10 billion (Satya et al., 2012). Internationally, China earns about US$130 million every year from the export of edible bamboo shoots, with US imports from China at around 4,400,000kg, accounting for 14.5% of the world’s total imports (Satya et al., 2012). Every year, the United States imports 30,000,000kg of canned bamboo shoots, and the annual *per capita* consumption of bamboo shoots can reach up to 3kg per person in Japan. However, consumption of bamboo shoots only accounts for 40% of the total fresh-shoot production, primarily because of its short shelf life (Quan and Liu, 2013). Most shoots are wasted or grow rapidly into mature culms in the field and lose their edible and commercial value.

Bamboo shoots are usually harvested with a spade or hoe, which can leave a severe wound at the cut end (Kleinhenz et al., 2000). Therefore, bamboo shoots deteriorate rapidly from mechanical injury during harvest, resulting in significant loss of quality (Li et al., 2019a). The degradation of nutrients and interstitial and woody fibers leads to physical and chemical changes in fresh bamboo shoots, which, in turn, negatively limit the shelf life during storage and affect consumer preferences (Pongprasert et al., 2007). The perishable nature of bamboo shoots is a major challenge during postharvest storage. To date, research has focused on the preservation technology for bamboo shoots. Hua (1987) reported that low temperatures and packaging techniques can reduce transpiration efficiently for long-distance transport of bamboo shoots. Cheng (2006) reported that vacuum cooling techniques can increase the cooling speed and prolong the preservation period of bamboo shoots during storage. Wang (2002) reported that modified atmospheric packaging is effective in preventing browning and lignification of bamboo shoots. The optimum temperature and packaging conditions for bamboo shoot storage are recommended at 1°C for semipermeable materials, which can extend the shelf life beyond 28d (Kleinhenz and Midmore, 2002). Moreover, nitric oxide (NO) and gamma irradiation techniques have been applied to the postharvest storage of fresh shoots, achieving some good results (Yang et al., 2010; Wang et al., 2019).

Because of the complexity of bamboo shoot growth, there are currently no efficient methods that completely inhibit quality deterioration and senescence of postharvest shoots, which correlate with the postharvest physiology of the bamboo shoots. However, no systematic work has, to our knowledge, been focused on the postharvest physiology of bamboo shoots. Such a systematic study of the physiological mechanisms for postharvest deterioration in bamboo shoots is needed.

The deterioration of quality in bamboo shoots after harvest has mainly been characterized by weight loss, high respiration, browning symptoms, fungal infections, and lignification (Bal et al., 2012; Wang et al., 2017). Respiration is the primary metabolism effect on the shelf life of harvested fruit and vegetables and on their quality and nutritional changes during storage. Continuous respiration can result in quality deterioration and nutrient reduction, including carbohydrates and proteins (Bal et al., 2012). Liu (1992) also attributed the loss of quality in bamboo shoots to high respiration rates. Respiration accounts for a large percentage of total weight loss (Kleinhenz et al., 2000). Moreover, fiber content in bamboo can increase quickly from the cut end toward the tip during storage, and phenylalanine ammonia lyase (PAL) activity also increases after harvest, which closely correlates with the increases in crude fiber and lignin (Chen et al., 1989). All these physiological changes can deteriorate fresh-shoot quality during postharvest storage, reducing commercial value and limiting long-distance transport.

To develop more-effective preservation techniques for fresh bamboo shoot storage, the basic postharvest physiology of bamboo shoots needs to be ascertained.

*Fargesia yunnanensis* is an alpine bamboo species, primarily found in Sichuan and Yunnan provinces of China (Wang et al., 2009). It is one of the most easily available resources for supplementing local wood and is also known for its delicious shoots. Its shoot yield can reach as much as 4,500kghm^−2^ without management, which will mostly become culms in the field because of the lack of effective processing technologies. In this study, the browning, weight loss, lignification, fibrosis, and nutrient degradation of *F. yunnanensis* shoots were evaluated during postharvest storage, and their physiological and biochemical changes under various storage conditions were analyzed. The results can provide better theoretical information for postharvest processing and storage of fresh bamboo shoots. Currently, cold-chain transportation is extensively employed in the storage and transportation of fresh fruit and vegetables to extend storage life and to retain process quality. That technique is also economically feasible for the commercial storage of bamboo shoots. Therefore, it is essential that the preservation effects of low-temperature storage techniques on bamboo shoots be analyzed, and their morphological and physiological changes and related theoretical aspects be evaluated so as to improve recommendations for maximizing process quality of postharvest bamboo shoots during storage.

## Materials and Methods

### Plant Material

According to the growth regularity of *F. yunnanensis* (Wang et al., 2016), fresh shoots were harvested from a plantation in the Xishan Mountain (100°202*E, 25°562*N; altitude~2,000m), in Kunming City, Yunnan Province, China. The fresh shoots used for samples had no damage, deformities, disease, insects or pests, and no shrinkage. Approximately 60–80cm of the bamboo shoot root was unearthed. The shoots were washed and stored at 4°C in a car refrigerator and immediately transported to the laboratory within 2h.

A total of 30 fresh shoots were stored at 25°C [determination of 25°C under room temperature storage (RT25)] and at 4°C [determination of 25°C under cold temperature storage (CT25) and determination of 4°C under cold temperature storage (CT4)]. Each treatment consisted of five groups, with three shoots in each group. The shoots stored at different temperatures were sampled every 3d.

After shoot sheaths were stripped, the sampled shoots were divided into upper, middle, and bottom portions of the plant according to their length. The divided samples were then cut into small strips with a sharp razor. For anatomical characterization, the samples were soaked in a formalin-acetic acid (FAA) fixative solution (1.85% formaldehyde+45% alcohol+0.25% acetic acid), and air was removed with a vacuum pump. For physiological and biochemical analysis, the samples were immediately frozen and stored in liquid nitrogen.

### Fresh Weight Loss and Respiration Rates During Storage

The weights of the shoots were recorded after various storage times. The fresh-weight loss was calculated according to the equation based on Pongprasert et al. (2007): Weight loss=[(Fresh weight−Weight after storage)/Fresh weight], which was expressed as gkg^−1^.

Respiration rates from the shoot sheaths and the shoots were measured with a portable photosynthesis system (Li6400 XT; Li-Cor, Lincoln, NE, United States). The shoot sheaths were cut off, and the shoots were sliced to 1cm×5cm×1cm in length, width, and thickness for the measurement of respiration rates. Different parts of the shoots were measured and compared under different storage conditions.

### Browning Analysis

The browning of bamboo shoots was observed and recorded every 3d during storage. The browning rate was calculated according to the equation: Browning rate=(Fresh weight of the browning parts/Fresh total weight), which was expressed as gkg^−1^.

### Localization of Starch Grain

After fixing in the FAA solution, the samples were cut into 7-μm-thick paraffin sections with a rotary microtome (Leica, Wetzlar, Germany). Starch grains in the shoots were localized with a periodic acid–Schiff (PAS) reaction (Chu, 1963). The paraffin sections were soaked in 0.5% KIO_4_ for 10min, followed by 30min in a Schiff reagent, then dehydrated in a graded series of ethanol, and stained with Fast Green FCF (Ameresco 0689; Solarbio Life Science, Beijing, China). The sections were observed under a light microscope (E400; Nikon, Tokyo, Japan).

### Assay and Content Determination

#### Soluble Sugar, Starch, and NSC Contents

The total soluble sugar content in the shoot samples was measured according to the Chong et al. (2007) method: 2g of fresh shoot tissue from each sample was fully milled in liquid nitrogen and, then, extracted with 10ml of deionized water at 70°C. The extracts were centrifuged at 11,370×*g* for 20min, and the supernatants were collected for determination of the soluble sugar content. The sediments were collected and used for the subsequent determination of the starch content. The supernatants were freeze-dried and redissolved in 1ml deionized water. All samples were measured three times.

The nonstructural carbohydrate (NSC) pool is the sum of soluble sugars and starches in plants (Richardson et al., 2013). The NSC content of the bamboo shoots was calculated based on the total content of soluble sugar and starch during storage, using Office 2010 software (Microsoft Corporation, Redmond, WA, United States).

#### Soluble Protein and Free Amino Acid Content

The soluble protein content was measured by the Coomassie brilliant blue G-250 method (Lott et al., 1983). A 1.0-g sample of dried bamboo shoots was ground with 5ml of phosphoric acid buffer at 0.1molL^−1^ (pH 7.0), which was then centrifuged at 1260×*g* for 10min at 4°C. Next, 0.2ml of the supernatant was diluted into a 1-ml solution volume. The soluble protein content was determined spectrophotometrically at 595nm.

The free amino acid content was measured with ninhydrin and ascorbic acid (Acharyulu et al., 2013). A 1.0-g dried sample was ground with 20ml of distilled water and incubated at 70°C for 30min. The solution was then filtered and diluted to a final volume of 25ml; from which, a 2-ml solution was centrifuged at 710×*g* for 5min at RT, and 0.5ml of the supernatant was diluted to a volume of 2ml by adding 1ml of water and 0.5ml chloroform. The solution was mixed intensively, and after stratification, 1ml of the supernatant was diluted to a volume of 4ml with the addition of 2.9ml of 0.1% ninhydrin and 0.1ml of 0.1% ascorbic acid, which was then incubated in a boiling water bath for 30min. The soluble protein content was determined spectrophotometrically at 570nm.

The total content of the soluble protein and free amino acids [soluble protein+amino acid (PA)] was calculated and recorded for the shoots during storage in Office 2010 software.

#### Sucrose- and Starch-Catabolizing Enzymes

Extraction of crude enzymes was conducted according to Moscatello et al. (2011). Briefly, to extract the enzymes from the bamboo shoots, 0.5*g* of each fresh sample was added to 3ml of the extraction buffer (precooled at 4°C), which contained 50mmolL^−1^ HEPES/NaOH (pH 7.5), 7.5mmolL^−1^ MgCl_2_, 1mmolL^−1^ EDTA (ethylenediaminetetraacetic acid), 2% PEG4000, 2% PVP (polyvinyl pyrrolidone), and 5mmolL^−1^ DTT (1,4-ditjiothreitol), and the mixture was homogenized for 1min. The homogenate was centrifuged at 4°C at 3760×*g* for 10min. The supernatant was diluted to 1.5ml for the enzyme solution, which was then stored at 4°C for further enzyme assays.

The activity of the soluble acid invertase (SAI) and the insoluble extracellular invertase (CWI) was assessed as described by Wang et al. (2019). The change in sucrose synthase (SUSY) was measured as described by Sung et al. (2006).

Starch phosphorylase (STP) activity was measured according to the Wang et al. (2019) method. To extract the enzyme, 1g of fresh shoot was added to 2ml of extraction buffer (precooled to 4°C), which contained 5mmolL^−1^ MgCl_2_, 1mmolL^−1^ EDTA, 2% PVP, 0.1% BSA (bovine serum albumin), and 5mmolL^−1^ DTT, and the mixture was homogenized for 1min. The homogenate was centrifuged at 4°C at a rate of 3,760×*g* for 10min. The supernatant was stored at −20°C. For the STP activity assay, the 1-ml reaction system used 50mmolL^−1^ HEPES (pH 7.0), 0.1% soluble starch, 10mmolL^−1^ sodium phosphate, 0.4mmolL^−1^ NAD (nicotinamide adenine dinucleotide), 0.1% BSA, 1U phosphoglucose mutase, and 2U 6-phosphoglucose dehydrogenase, which were added to a test tube, and then, 50μl of the enzyme extract was added. The final solution was incubated at 25°C for 30min. Absorbance at 340nm was immediately measured and used as the NADH (reduced NAD) value for the reaction.

#### PAL, POD, and PPO Activity

To extract enzymes from the shoots, 3g each of fresh sample was homogenized in 9ml of sodium borate buffer (pH 8.8; 1g polyvinylpolypyrrolidone, 5mm β-mercaptoethanol, and 2mm EDTA), and the mixture was homogenized at 5050×*g* for 1min. The homogenate was centrifuged at 4°C at a rate of 11,370×*g* for 15min. The supernatant was stored at 4°C for further enzymatic activity measurement.

Phenylalanine ammonia lyase activity was measured according to Gao (2006): 300μl of 50mm boric acid buffer (pH 8.0) with 20mm l-phenylalanine was added to 300μl of supernatant in a test tube, and then, 600μl of distilled water was added. As a control, 900μl of distilled water was added without the buffer. The reactions were incubated at 4°C and 25°C for 90min and were terminated by boiling at 100°C for 10min. The absorbance was immediately measured at 290nm.

Peroxidase (POD) activity was measured as described by Aquino-Bolaños and Silva (2004) and Luo et al. (2010). Polyphenol oxidase (PPO) activity was measured according to the method described by Demeke et al. (2010).

#### Malondialdehyde, Hydrogen Peroxide, and Total Phenol Content

Malondialdehyde (MDA) content in the bamboo shoots during storage was measured according to the Davey et al. (2005) method. Hydrogen peroxide (H_2_O_2_) content was evaluated as described by Rendón et al. (2013). Total phenol content was measured according to the method of Shen et al. (2006). A 1-*g* sample of dried shoot was mixed with 10ml ethanol in hydrochloric acid buffer (50% ethanol and 1% hydrochloric acid) and incubated at 4°C for 24h. The solution was then filtered and diluted to a final volume of 100ml, and the absorbance was measured at 280nm.

### Cinnamic Acid, Lignin, and Cellulose Content

Cinnamic acid content was determined with a high-performance liquid chromatography (HPLC) system (Agilent 1100; Agilent Scientific Instruments, Santa Clara, CA, United States) according to the method of Sipkina and Skorik (2015) with some modifications. Dried shoot tissue (3*g*) was homogenized in liquid nitrogen, and then, a 10-μl aliquot of each sample was injected into the HPLC system, equipped with a Zorbax SB-C18 column (150×4.6mm; MilliporeSigma, Burlington, MA, United States) with a particle size of 5μm.

Lignin was measured as described by Liu (2003): 1g of the dried shoot tissue was weighed and homogenized in 5ml of 95% alcohol. The homogenate was centrifuged at 710×*g* for 7min, and the sediment was washed three times with a solution of alcohol and hexane (volume ratio, 1:2). The sediment was dried and redissolved in glacial acetic acid solution containing 25% acetyl bromide. The reaction mixture was incubated at 70°C for 30min, which was terminated by adding 0.9ml of 2molL^−1^ NaOH, followed by 5ml of glacial acetic acid and 0.1ml of 7.5molL^−1^ hydroxylammonium chloride for a final volume of 10ml. That final solution was recentrifuged at 710×*g* for 7min, and the supernatant absorbance was measured at 280nm.

The cellulose content in the bamboo shoots was measured according to the methods of Gao (2006) and Chen et al. (2013) with some modifications. A 0.2-g dried sample was extracted in 60ml of 60% (v/v) H_2_SO_4_ for 0.5h in an ice bath, followed by the addition of 60% (v/v) H_2_SO_4_ for a final volume of 100ml. The mixture was shaken and filtered in a breaker, and 2ml was removed in a test tube to which 0.5ml of 2% anthrone reagent and 5ml H_2_SO_4_ were added. The final solution was shaken and allowed to stand for 0.5h, and the supernatant was measured to determine the cellulose content at 620nm, using anthrone as a color reagent and glucose as the quantification standard.

### Statistical Analysis

All measurements in this study used three replicate samples, and each sample was determined three times. The data were tested by analysis of variance using SPSS 17 software (IBM, Chicago, IL, United States). Least-significance differences were employed to compare significance between means at the 5% level. Experimental data were analyzed to determine significant differences in the measurements of shoots stored at different conditions and the same condition for 0, 3, 6, 9, and 12d. The various values for the content of NSC, PA, lignin and cellulose, total phenols, and respiration rates were calculated in Office 10. The correlation analyses of the physiological indexes during storage were conducted in SPSS 17.

## Results

### Morphological Changes and Fresh Weight Loss During Storage

**Figure 1:**
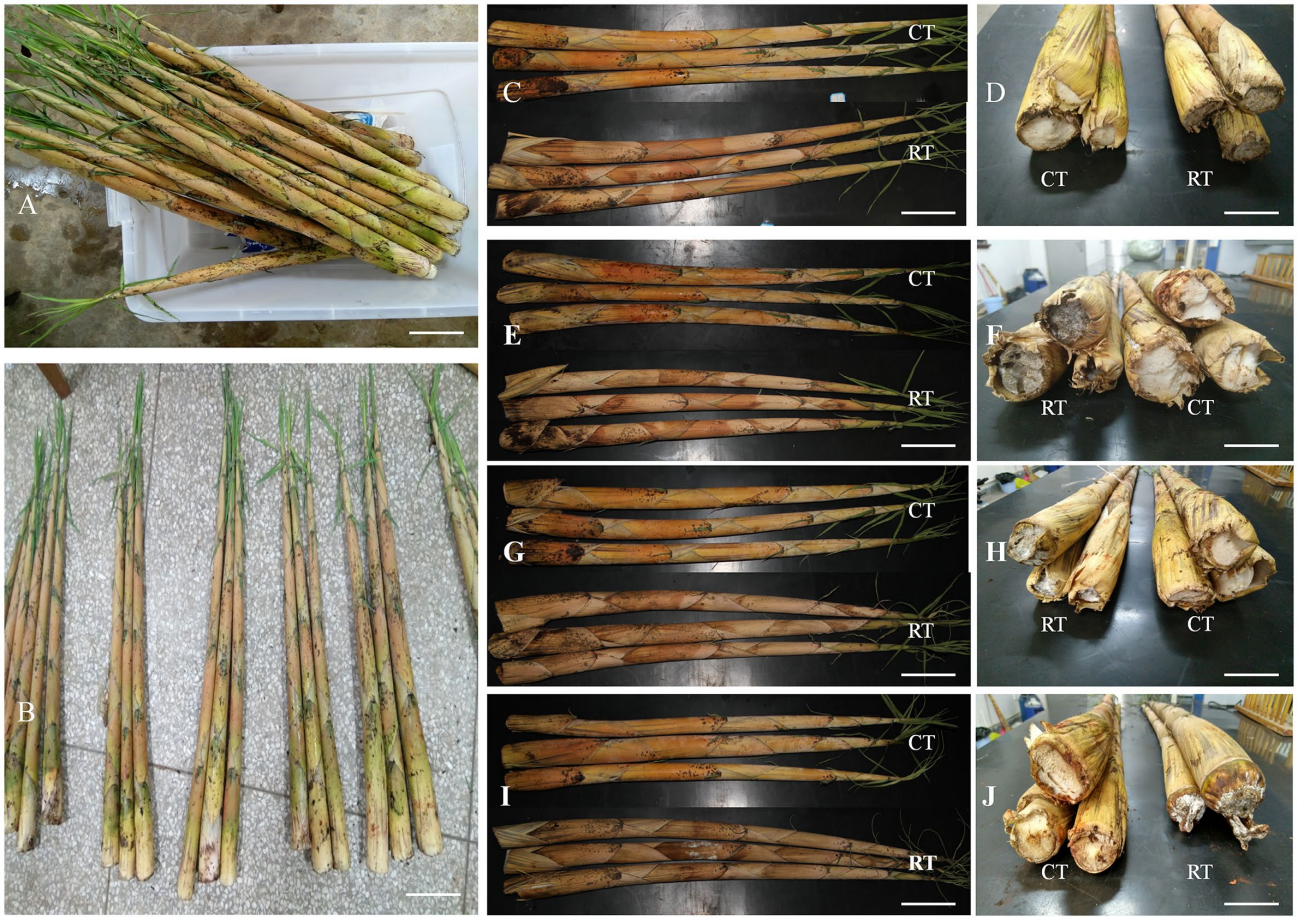
Appearance changes in *Fargesia yunnanensis* shoots under different storage conditions. **(A,B)** Fresh-cut shoots were divided into different groups as samples. **(C,D)** Differences in the appearance of the shoots stored at 25°C and 4°C after 3d. **(E,F)** Differences in the appearance of the shoots stored at 25°C and 4°C after 6d. **(G,H)** Differences in the appearance of the shoots stored at 25°C and 4°C after 9d. **(I,J)** Differences in the appearance of the shoots stored at 25°C and 4°C after 12d. RT, bamboo shoots stored at room temperature (25°C); CT, bamboo shoots stored at cold temperatures (4°C); RT25, bamboo shoots stored at RT and analyzed at 25°C; and CT25, bamboo shoots stored at cold temperatures and analyzed at 25°C. Scale bar=6cm.

The fresh-cut shoots were stored at 25°C and 4°C for a storage period of 12d (Figures 1A,B). The sheaths at the bottom parts of the shoots began drying and shrinking under both storage temperatures after 3d (Figures 1C,D), and the shoots stored at 25°C darkened more than those stored at 4°C (Figure 1D). After a storage period of 6 and 9d, the shoot sheaths stored at room temperature (RT) had dried and shrunken significantly, and their cut surface became mildewed, whereas the shoots stored at 4°C remained fresh at the cut surface (Figures 1E–H). Moreover, the cut surfaces of the shoots stored at 4°C were still white and fresh after 12d, whereas there was obvious shrinkage and a covering of white fungus on shoots stored at RT (Figure 1J).

**Figure 2:**
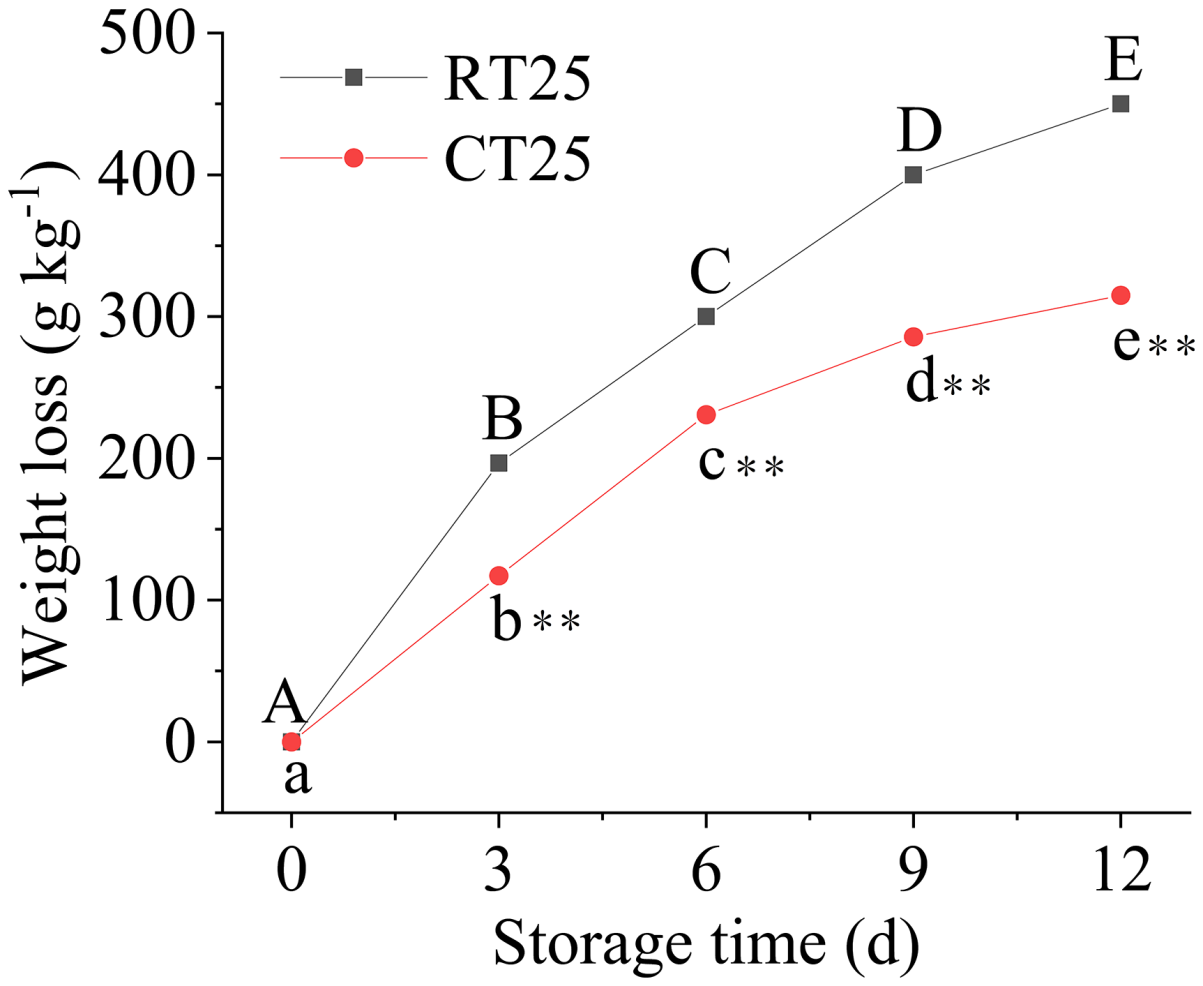
Fresh-weight loss percentage in the postharvest shoots stored at 25°C and 4°C. Data are presented as mean±standard deviation (*n*=3). Significant analysis was performed using the Student’s *t*-test, in which different letters represent significant difference between treatments within the group, and the asterisk represents the level of significance between groups (^*^means the difference is significant at the 0.05 level, ^**^means the difference is significant at the 0.01 level).

Fresh-weight loss from the shoots stored at 25°C and 4°C was evaluated every 3d (Figure 2). The shoots lost significant weight with storage time, whether at 25°C or 4°C. However, the fresh-weight loss always remained greater at 25°C than it did at 4°C, indicating that low-temperature storage can effectively attenuate weight loss, which accorded well with the morphological changes in the bamboo shoots stored at the different temperatures.

In addition, the shoots lost more of their weight during the first 3d than they did later in storage at 25°C (Figure 2), a loss that reached 197gkg^−1^. However, the weight loss increased consistently by 103gkg^−1^ during the second 3-d period and lost another 100gkg^−1^ during the third 3-d and another 150gkg^−1^ during the fourth 3-d periods. These results revealed that the greatest weight loss occurred primarily during the first storage period at RT. For bamboo shoots stored at 4°C, more weight was lost during the second 3-d storage period (a loss of 113gkg^−1^) and was far less than the weight lost by shoots stored at RT. Therefore, low-temperature storage effectively attenuates weight loss in bamboo shoots during storage.

### Respiration Rates of Shoots and Sheaths

**Figure 3:**
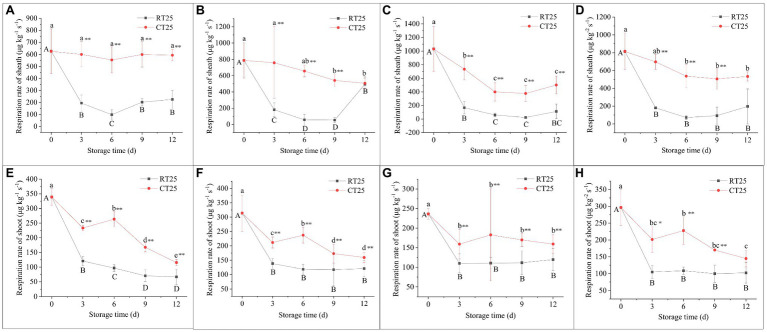
Respiration rates in bamboo shoot sheaths and shoots. **(A–D)** Respiration rates of sheaths under different storage conditions. **(E–H)** Respiration rates of shoots under different storage conditions. **(A)** Respiration rates of the sheaths in the upper plant parts of the bamboo shoots during storage at 25°C and 4°C. **(B)** Respiration rates of the sheaths in the middle plant parts of the bamboo shoots during storage at 25°C and 4°C. **(C)** Respiration rates of the sheaths in the bottom plant parts of the bamboo shoots during storage at 25°C and 4°C. **(D)** Mean respiration rates of the sheaths in all parts of the bamboo shoots during storage at 25°C and 4°C. **(E)** Respiration rates of the upper parts of bamboo shoots during storage at 25°C and 4°C. **(F)** Respiration rates in the middle plant parts of the bamboo shoots during storage at 25°C and 4°C. **(G)** Respiration rates in the bottom plant parts of the bamboo shoots during storage at 25°C and 4°C. **(H)** Mean respiration rates in all plant parts of the bamboo shoots during storage 25°C and 4°C. RT25, bamboo shoots stored at room temperature and analyzed at 25°C and CT25, bamboo shoots stored at cold temperatures and analyzed at 25°C. Data are presented as mean±standard deviation (*n*=3). Significant analysis was performed using the Student’s *t*-test, in which different letters represent significant difference between treatments within the group, and the asterisk represents the level of significance between groups (^*^means the difference is significant at the 0.05 level, ^**^means the difference is significant at the 0.01 level).

Respiration rates for the shoots and shoot sheaths during storage were also measured every 3d (Figure 3). The respiration rate of the bamboo shoots was expressed by determining the rate at the shoot skins.

The shoot sheaths stored at both 25°C and 4°C showed a decreasing trend in the order of the respiration rate from the bottom > middle > upper plant parts, which was probably due to their different degrees of development. Moreover, all sheaths showed a sharp decline in respiration rate during the first and second 3-d storage periods at 25°C, which then increased slightly during the subsequent storage periods (Figures 3A–C). Shoot sheaths stored at 4°C showed a similar trend but with higher respiration rates than the shoots stored at RT (Figure 3). These results show that shoot sheaths stored at low-temperature maintain higher respiration rates during storage.

The shoots had similar trends in respiration rates during storage at both 25°C and 4°C (Figures 3E–H). The respiration rates for all parts of the shoots declined sharply during the first 3-d storage period and increased during the second 3-d storage period, and then declined consistently, whether stored at 4°C or 25°C. In addition, all bamboo shoots stored at 4°C showed higher respiration rate values than those stored at 25°C, similar to the results for shoot sheaths. The upper and middle parts of the bamboo shoots showed higher respiration rates than did the bottom parts during storage, which was significantly different from the results for shoot sheaths.

### Dynamic Changes in Browning Rates During Storage

**Figure 4:**
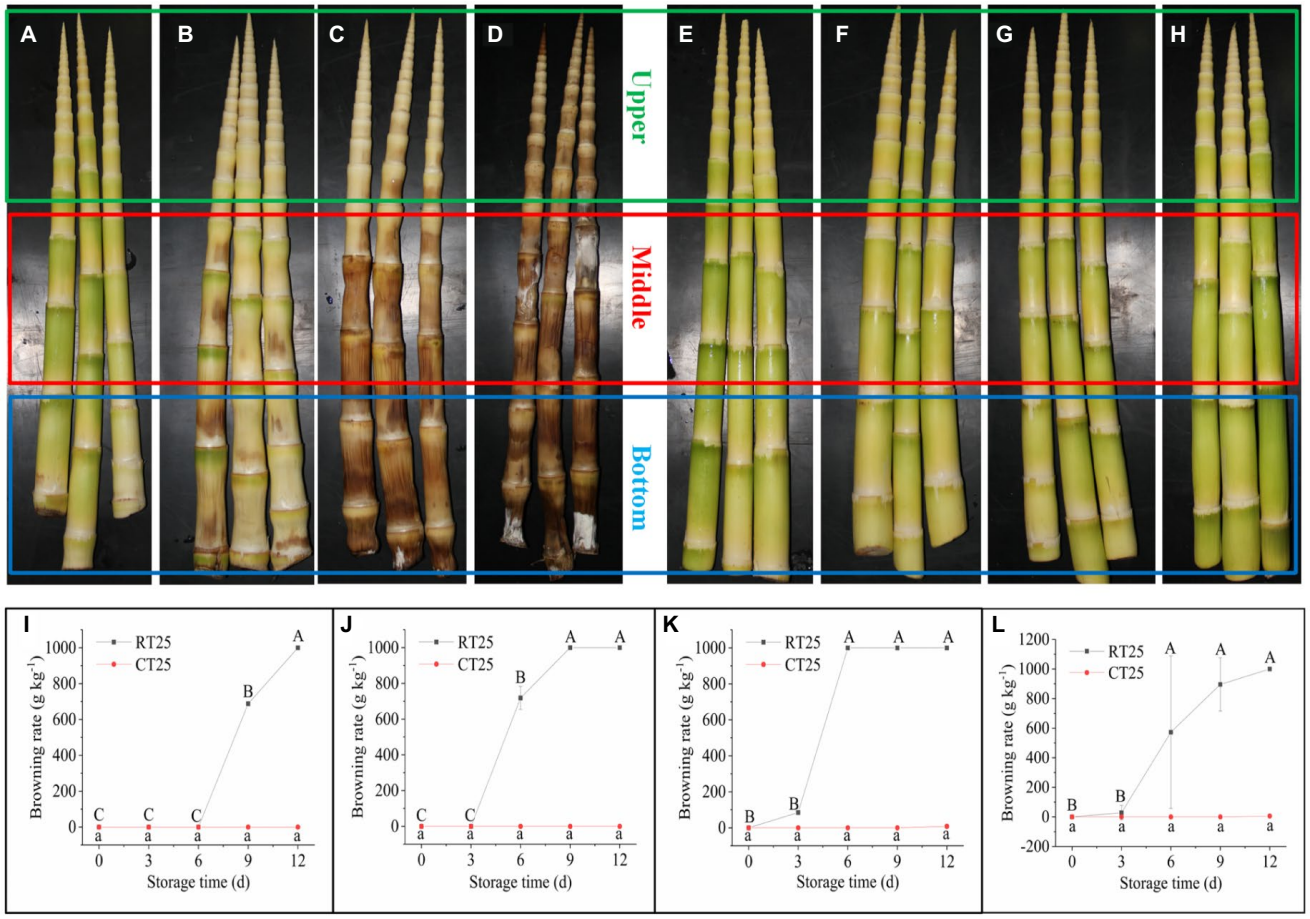
Shoot browning during storage at 25°C and 4°C. RT25, bamboo shoots stored at room temperature and analyzed at 25°C; CT25, bamboo shoots stored at cold temperatures and analyzed at 25°C. **(A–D)** The postharvest bamboo shoots browned rapidly at 25°C for shoots stored for 3 **(A)**, 6 **(B)**, 9 **(C)**, and 12 **(D)** d. **(E–H)** The postharvest bamboo shoots stored at 4°C, which retarded their browning for shoots stored for 3 **(E)**, 6 **(F)**, 9 **(G)**, and 12 **(H)** d. **(I–L)** Dynamic changes in browning rates of bamboo shoots during storage for upper **(I)**, middle **(J)**, and bottom **(K)** plant parts, and mean values **(L)**.

Bamboo shoots browned rapidly when stored at RT conditions (Figures 4A–D). Browning usually started from the bottom of the shoot and soon spread to the top. After 3d, a small part of the internode close to the nodal bridge in the bottom part began browning (Figure 4A), and then, the browned area expanded rapidly. After 6d, most parts of the middle and bottom of the shoot began browning (Figure 4B), and the bottom and middle parts were almost completely brown after 9d (Figure 4C). Subsequently, the entire shoot turned completely brown, and some of the internodes became moldy (Figure 4D). Therefore, the internodal part closest to the nodal bridge appears to be where browning first occurs (Figures 4A,B). For shoots stored under CT conditions, almost no samples turned brown, and no internodes became moldy during the entire storage period (Figures 4E–H).

We calculated the browning ratio of bamboo shoots during storage based on the weights of the browned parts. The upper parts of the bamboo shoots under RT conditions began browning during the third 3d of storage, with a browning rate of 688gkg^−1^, and were completely brown after 12d (Figure 4I). For the middle shoot parts, the internodes began browning during the second 3d of storage, with a browning rate of 718gkg^−1^, and were completely brown after 9d (Figure 4J). The browning rate of the bottom shoot parts reached 85gkg^−1^ after 3d and were completely brown after 6d. However, the browning rate of bamboo shoots stored under CT conditions was only 8gkg^−1^ after 12d (Figure 4K). The shoots stored at RT browned more easily than those stored under low temperatures (Figure 4L). Therefore, low temperatures significantly prevent the browning of bamboo shoots during storage.

### Dynamic Changes in Starch, Soluble Sugars, and NSC Content

**Figure 5:**
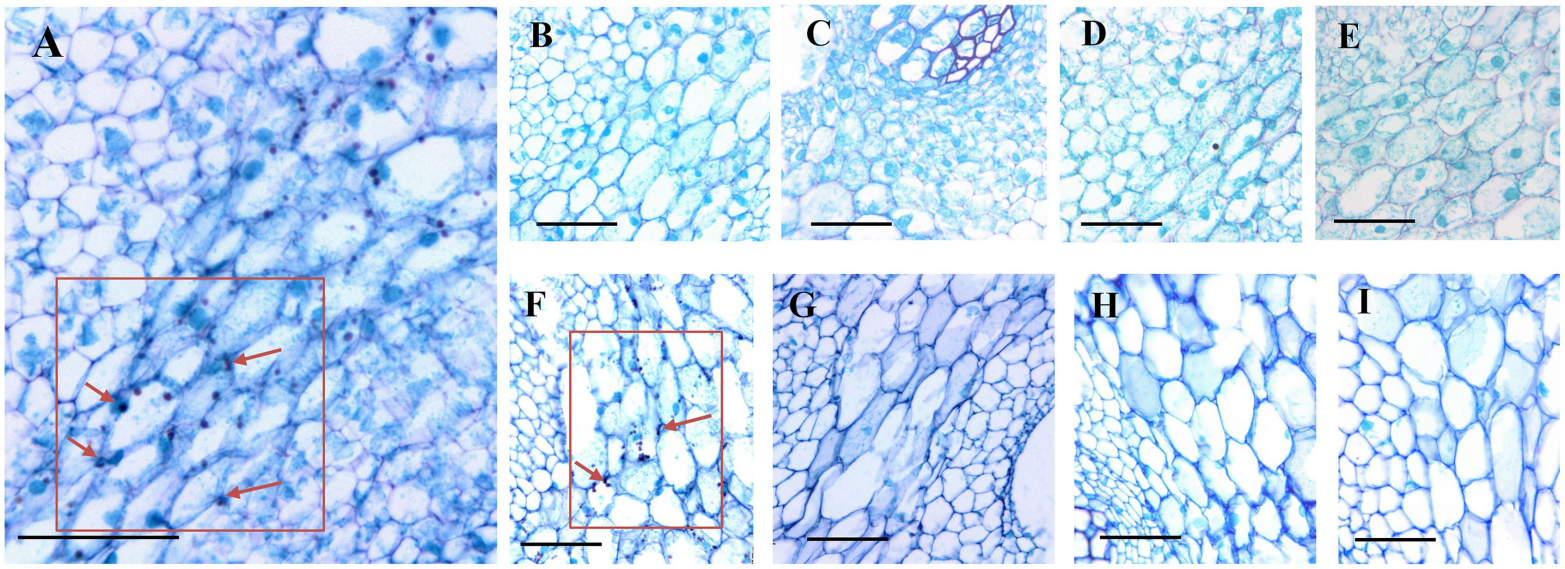
Dynamics of starch grains in postharvest bamboo shoots during storage at 25°C and 4°C. Scale bar=50μm. **(A)** Starch grains in the fresh-cut shoots. **(B–E)** Dynamics of starch grains in postharvest bamboo shoots during storage for 3 **(B)**, 6 **(C)**, 9 **(D)**, and 12 **(E)** d at 25°C. **(F–I)** Dynamics of starch grains in postharvest bamboo shoots during storage for 3 **(F)**, 6 **(G)**, 9 **(H)**, and 12 **(I)** d at 4°C. The arrow points to the starch granule.

According to the PAS reaction, many starch grains were localized in the parenchyma cells of the fresh-cut bamboo shoots (Figure 5A), but for shoots stored at 25°C, few starch grains remained after 3d (Figures 5B–E). Starch grains could still be observed in shoots stored at 4°C after 3d (Figure 5F) but not after longer storage periods (Figures 5G–I). This means that shoot starch grains were catabolized rapidly during the first 3d of storage at RT but were maintained under cold storage temperatures.

**Figure 6:**
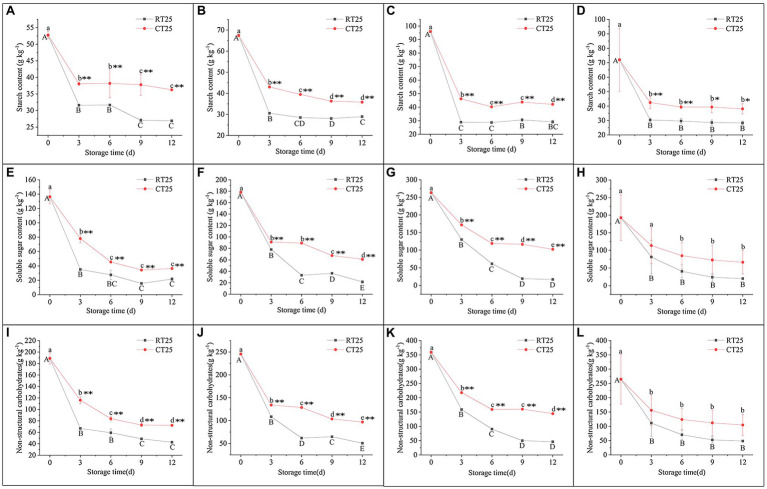
Dynamic changes in the content of starch, soluble sugar, and nonstructural carbohydrate (NSC) in bamboo shoots during storage at 25°C and 4°C. **(A–D)** Dynamic changes in starch content in shoots during storage for upper **(A)**, middle **(B)**, and bottom **(C)** plant parts, and mean values of all plant parts in bamboo shoots under different treatment conditions **(D). (E–H)** Dynamic changes in soluble sugar content in shoots during storage for upper **(E)**, middle **(F)**, and bottom **(G)** plant parts, and mean values for all plant parts in the bamboo shoots **(H). (I–L)** Dynamic changes in NSC content in shoots during storage for upper **(I)**, middle **(J)**, and bottom **(K)** plant parts, and mean values for all plant parts in the bamboo shoots **(L)**. Data are presented as mean±standard deviation (*n*=3). Significant analysis was performed using the Student’s *t*-test, in which different letters represent significant difference between treatments within the group, and the asterisk represents the level of significance between groups (^*^means the difference is significant at the 0.05 level, ^**^means the difference is significant at the 0.01 level).

The dynamic change in starch content was similar to the localization of starch grains during storage (Figures 6A–D). The starch content in all three parts of the shoot decreased sharply during the first 3d of storage and then consistently decreased during subsequent storage at RT and in cold temperature (CT) conditions. Compared with shoots stored at 25°C, those stored at 4°C always contained more starch during all storage periods, showing its inhibitory effects on starch degradation at CT.

The soluble sugar content in all parts of the shoots decreased sharply during the first 3d of storage and then consistently declined in subsequent storage periods (Figures 6E–H). The soluble sugar content declined more drastically than the starch content did. Similarly, the NSC content also decreased consistently during storage, and the decrease was greatest during the first 3d than it was in the following storage periods (Figures 6I–L).

#### Activity of Sucrose- and Starch-Catabolizing Enzymes

**Figure 7:**
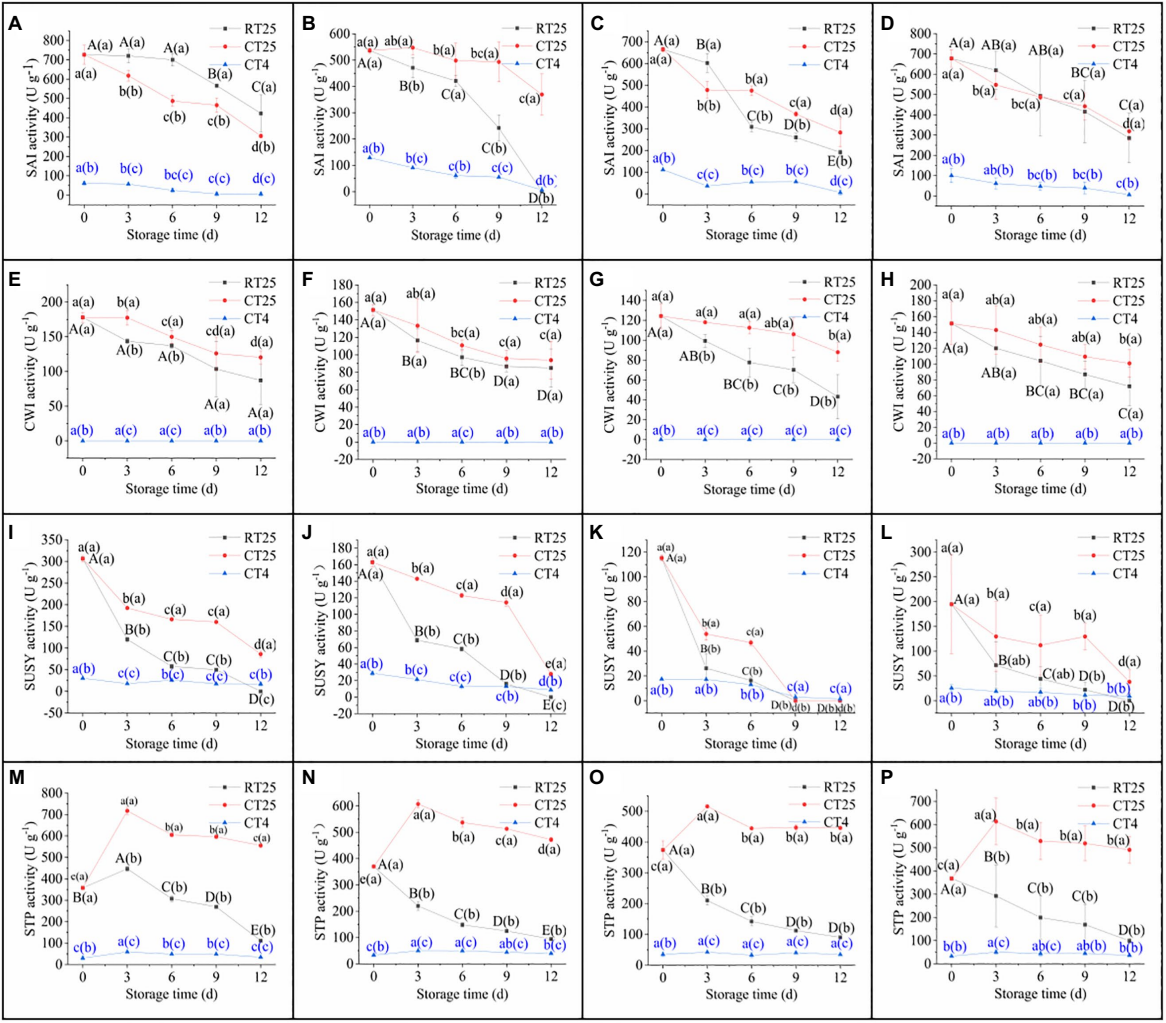
Activity of sucrose- and starch-catabolizing enzymes in bamboo shoots during storage at 25°C and 4°C. RT25, bamboo shoots stored at room temperature and analyzed at 25°C; CT25, bamboo shoots stored at cold temperatures and analyzed at 25°C; and CT4, bamboo shoots stored at cold temperatures and analyzed at 4°C. **(A–D)** Dynamic changes in SAI activity in shoots during storage for upper **(A)**, middle **(B)**, and bottom **(C)** plant parts, and mean values **(D). (E–H)** Dynamic changes in CWI activity in shoots during storage for upper **(E)**, bottom **(F)**, and bottom **(G)** plant parts, and mean values **(H). (I–L)** Dynamic changes in SUSY activities in shoots during storage for upper **(I)**, middle **(J)**, and bottom **(K)** plant parts, and mean values **(L). (M–P)** Dynamic changes in STP activity in shoots during storage for upper **(M)**, middle **(N)**, and bottom **(O)** plant parts, and mean values **(P)**. Data are presented as mean±standard deviation (*n*=3). Significant analysis was performed using the Student’s *t*-test, in which different letters represent significant difference between treatments within the group, and the different letters in brackets represent the level of significance between groups (see also of Figure 10).

Sucrose and starch catabolism in bamboo shoots during storage was determined by the changes in SAI, CWI, SUSY, and STP every 3d (Figure 7). To analyze the effects of CT on enzymatic activities, all shoot samples stored at 4°C were measured separately at CT25 and CT4.

Under RT storage conditions, the SAI activity decreased slightly in the upper parts of the shoots during the first 6-d period but decreased significantly in subsequent storage periods (Figure 7A). In addition, SAI decreased significant in the middle and bottom shoot parts during the first 6d of storage (Figures 7B,C). The shoots stored at 4°C, whether measured at 25°C or at 4°C, also showed a consistent decrease with storage time. The enzymatic activity determined at 25°C was closer to those of shoots stored at 25°C but was far greater than that of those stored at 4°C (Figure 7D). These results indicate that enzymatic activity was significantly inhibited under CT conditions and recovered rapidly during subsequent RT conditions.

Similarly, CWI decreased consistently in all parts of the shoots during storage (Figures 7E–H). Moreover, CWI activity was always greater in shoot samples stored at 4°C than it was in those stored at 25°C. When the shoot samples stored at 4°C were analyzed at 4°C, the CWI activity was too low to be detected, which implies that CWI activity in bamboo shoots is well maintained but deeply inhibited under CT storage conditions. In addition, the CWI activity was less than that of SAI.

Sucrose synthase activity decreased sharply during the first 3d of storage and then gradually decreased subsequently in all parts of the shoot (Figures 7I–L). The SUSY activity of shoot samples stored under CT condition showed a similar trend, with higher values when determined at 25°C but lower values when determined directly at 4°C. Therefore, CT storage effectively inhibits, but maintains, SUSY activity, which recovers rapidly at RT.

STP activity was similar to that of SAI, CWI, and SUSY under RT and CT conditions during storage (Figures 7M–P). Under RT storage conditions, STP activity in the upper parts of bamboo shoots increased significantly during the first 3d and then decreased gradually, whereas, in the middle and bottom parts, the activity always decreased consistently with storage time. For shoots stored under CT conditions, the STP activity determined at 25°C increased significantly over the first 3d and then gradually decreased in subsequent days, but was always greater than that of shoots stored under RT conditions. The STP activity was also significantly inhibited under CT storage conditions.

#### Dynamic Changes in PA Content

**Figure 8:**
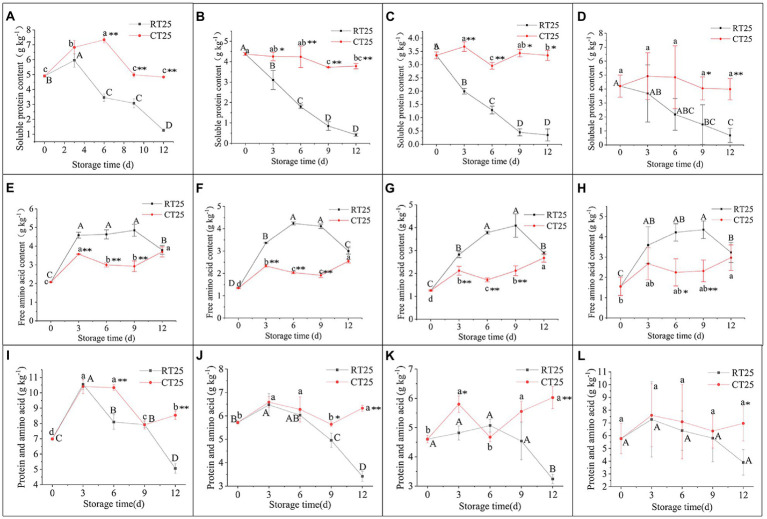
Dynamic changes in soluble protein, free amino acid, and PA (soluble protein and free amino acid) content in shoots stored at 25°C and 4°C. RT25, bamboo shoots stored at room temperature and analyzed at 25°C; CT25, bamboo shoots stored at cold temperature and analyzed at 25°C. **(A–D)** Soluble protein content in shoots during storage for upper **(A)**, middle **(B)**, and bottom **(C)** plant parts, and mean values **(D). (E–H)** Free amino acid content in shoots during storage for upper **(E)**, middle **(F)**, and bottom **(G)** plant parts, and mean values **(H). (I–L)** PA content in shoots during storage for upper **(I)**, middle **(J)**, and bottom **(K)** plant parts, and mean values **(L)**. Data are presented as mean±standard deviation (*n*=3). Significant analysis was performed using the Student’s *t*-test, in which different letters represent significant difference between treatments within the group, and the asterisk represents the level of significance between groups (^*^means the difference is significant at the 0.05 level, ^**^means the difference is significant at the 0.01 level).

The soluble protein content of shoots varied under RT and CT storage conditions (Figures 8A–D). In general, soluble protein decreased consistently with storage time in bamboo shoots stored at 25°C. In the upper shoot parts, the soluble protein increased over the first 3d and then decreased significantly (Figure 8A). In the middle and bottom shoot parts, the soluble protein decreased consistently with storage time. The shoots stored at 4°C, which showed more soluble protein in all parts than those stored at 25°C, had increased soluble protein at first and then decreased with storage time.

The free amino acid content in the upper, middle, and bottom parts of the shoots kept at RT increased during the first 9d of storage but decreased consistently during subsequent storage (Figures 8E–H), whereas for shoots stored at 4°C, the free amino acid content in the upper and middle shoot parts increased during the first 3d, decreased consistently during the second and third 3-d storage periods, and increased during the final 3d of storage (Figures 8E,G). The bottom shoot parts showed increased in amino acid content during the first 3d, decreased during the second 3d of storage, and increased consistently during subsequent storage periods (Figure 8F).

Generally, bamboo shoots stored at 4°C showed greater PA content than those stored at 25°C, which implies that low temperatures inhibit PA degradation and maintains shoots well during storage (Figures 8I–L).

#### Dynamic Changes in the Content of MDA, H_2_O_2_, and Total Phenols During Storage

**Figure 9:**
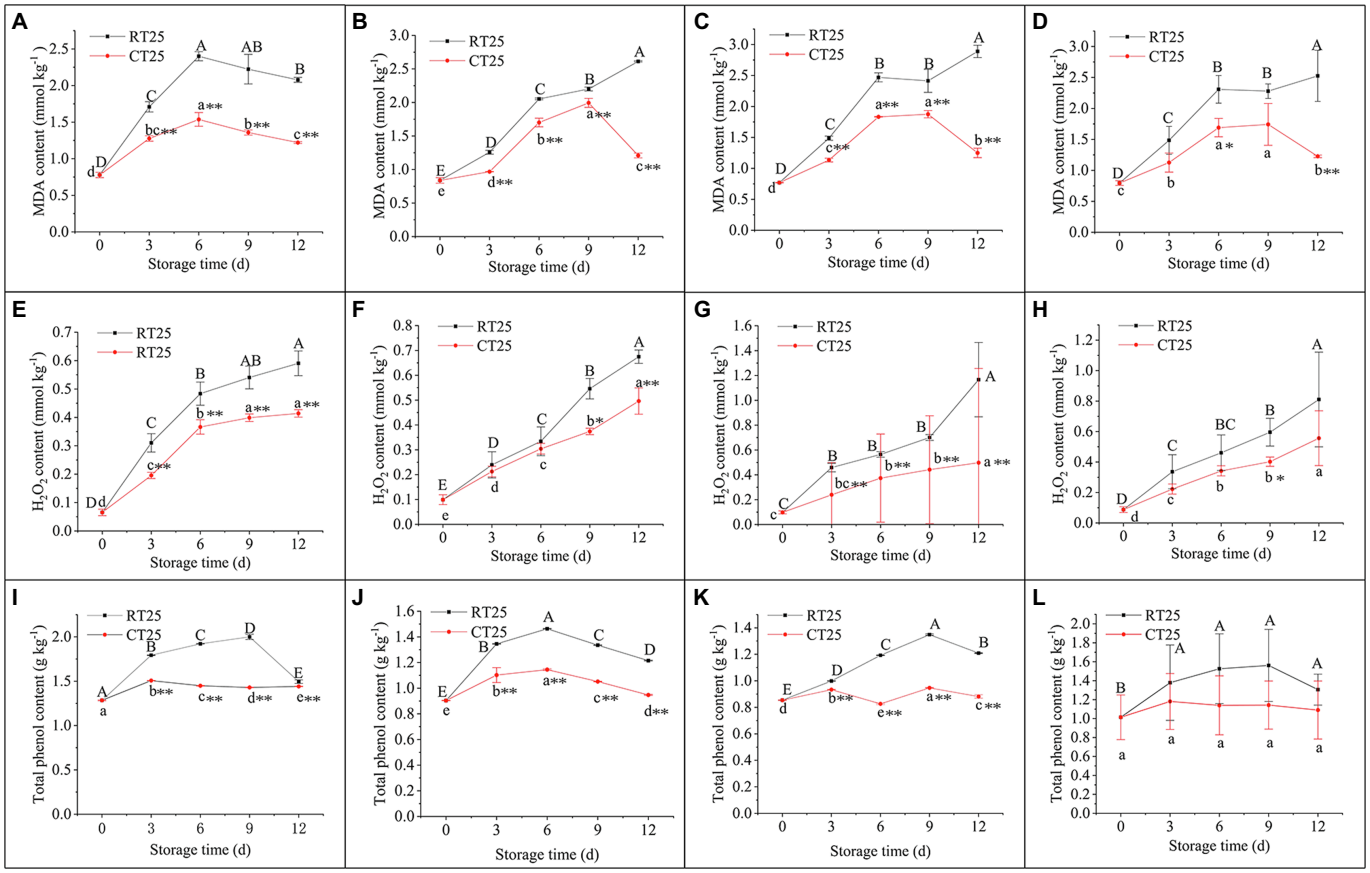
Dynamic changes in MDA, H_2_O_2_, and total phenol content in bamboo shoots after storage at 25°C and 4°C. RT25, bamboo shoots stored at room temperature and analyzed at 25°C; CT25, bamboo shoots stored at cold temperatures and analyzed at 25°C. **(A–D)** MDA content in shoots during storage for upper **(A)**, middle **(B)**, and bottom **(C)** plant parts, and mean values **(D). (E–H)** H_2_O_2_ content in shoots during storage for upper **(E)**, middle **(F)**, and bottom **(G)** plant parts, and mean values **(H). (I–L)** Total phenol content in shoots during storage for upper **(I)**, middle **(J)**, and bottom **(K)** plant parts, and mean values **(L)**. Data are presented as mean±standard deviation (*n*=3). Significant analysis was performed using the Student’s *t*-test, in which different letters represent significant difference between treatments within the group, and the asterisk represents the level of significance between groups (^*^means the difference is significant at the 0.05 level, ^**^means the difference is significant at the 0.01 level).

The dynamic changes in MDA, H_2_O_2_, and total phenol content during storage were determined for different parts of the bamboo shoots under RT and CT conditions. Under RT storage conditions, the MDA content increased sharply in the first 6d, decreased gradually in the upper parts, but increased continuously in the middle and bottom parts during the entire storage period (Figures 9A–D). For shoots stored in CT conditions, the MDA in the upper parts increased consistently during the first 6d and then decreased consistently (Figure 9A), whereas in the middle and bottom parts, the MDA contents increased consistently until the third storage period and then decreased significantly (Figures 9B,C). The accumulation of MDA was always less in shoots stored at 4°C than it was in those stored at 25°C, which indicates that low temperatures significantly reduce the production of MDA.

As shown in Figures 9E–H, H_2_O_2_ content in shoots stored at both 25°C and 4°C increased gradually with time. H_2_O_2_ content was always less in shoots stored at 4°C than it was in those stored at 25°C, and the difference in those values increased greatly with storage time.

Total phenols can be oxidized into quinones, which is an important substance in the browning process (Toivonen and Brummell, 2008; Deutch, 2018). Under RT conditions, total phenol content in the upper and bottom parts of the bamboo shoots increased consistently during the first 9-d period but then began decreasing. However, total phenol content in the middle part increased at first and then decreased significantly, with the highest values on day 6 (Figure 9J). For shoots stored at 4°C, a similar trend was observed in total phenol content, but the values were always less than those stored at 25°C (Figures 9I–L). Because, under RT conditions, the bottom parts of the shoots started browning in the first 3d, whereas their total phenol content was less in the upper shoot parts, total phenols may accumulate more slowly than the rate of oxidation, accounting for the rapid browning in the bottom parts of bamboo shoots during storage.

#### Dynamic Changes in PAL, POD, and PPO Activity During Storage

Bamboo shoots stored at 25°C and 4°C were both measured at RT to determine PAL, POD, and PPO activity, and the shoot samples stored at 4°C were also measured at 4°C to analyze the inhibitory effects of low temperature conditions on enzymatic activity in the shoots during storage.

**Figure 10:**
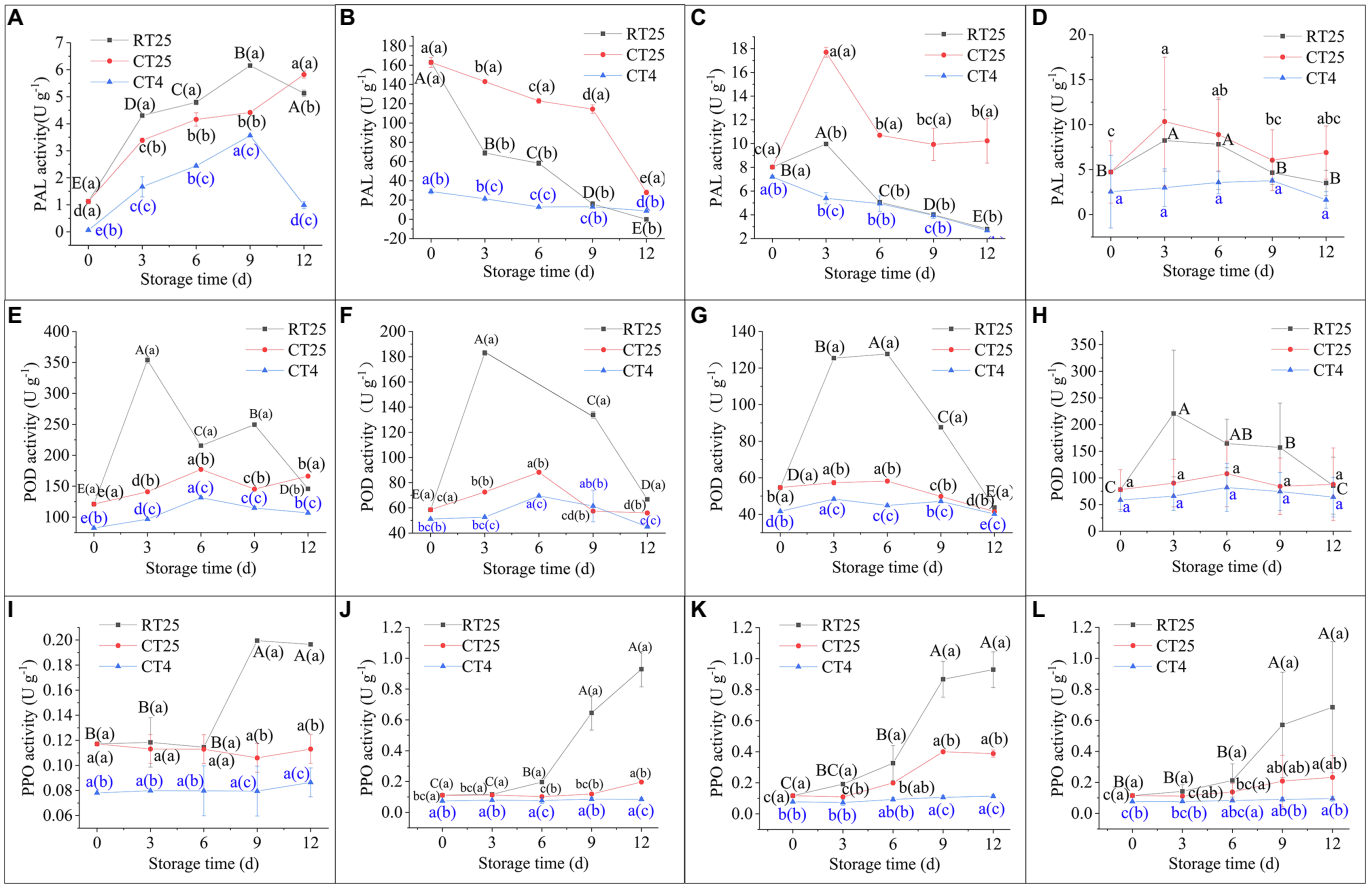
Dynamic changes in phenylalanine ammonia lyase (PAL), Peroxidase (POD), and Polyphenol oxidase (PPO) activities in bamboo shoots during storage at 25°C and 4°C. RT25, bamboo shoots stored at room temperature and analyzed at 25°C; CT25, bamboo shoots stored at cold temperatures and analyzed at 25°C; and CT4, bamboo shoots stored at cold temperatures and analyzed at 4°C. **(A–D)** PAL activity in shoots during storage for upper **(A)**, middle **(B)**, and bottom **(C)** plant parts, and mean values **(D). (E–H)** POD activity in shoots during storage from upper **(E)**, middle **(F)**, and bottom **(G)** plant parts, and mean values **(H). (I–L)** PPO activity in shoots during storage for upper **(I)**, middle **(J)**, and bottom **(K)** plant parts, and mean values **(L)**.

Under RT storage conditions, the various parts of the shoots showed similar PAL activity, which increased at first and then decreased significantly, with the highest values on the ninth day in the upper shoot parts but on the sixth and third days for the middle and bottom parts, respectively (Figures 10A–D). Their highest rates of enzymatic activity were greater in the bottom parts than they were in the upper and middle shoot parts, meaning that PAL activity increases more rapidly in the bottom than it does in other parts. The shoots stored under CT conditions showed a similar trend in activity. Their activity was close to that of the shoots stored under RT conditions when they were determined at 25°C but was much less when directly determined at 4°C, which indicates that the low temperature inhibits PAL activity in shoots during storage.

Peroxidase activity in bamboo shoots stored under RT and CT conditions showed a similar trend to that of PAL, also increasing initially and then decreasing consistently, with the highest values on the third or sixth days (Figures 10E–H). Unlike PAL, POD activity values for shoots stored under CT conditions and determined at 25°C were closer to those determined at 4°C, which implies PAL activity in bamboo shoots stored at 4°C does not completely recover under RT conditions. The low temperature well inhibits PAL activity in bamboo shoots during storage.

Similarly, PPO activity increased consistently with time and was greater in shoots stored at 25°C than it was in those stored at 4°C (Figures 10I–L). When determined at 4°C, PPO activity was much less than it was when determined at 25°C in the shoots stored at 4°C. In general, the activity values for POD were far greater than they were for PAL and PPO, and PAL had the least activity during storage. This result implies that POD has an important role in the process of senescence for bamboo shoots during storage.

#### Dynamic Changes in Cellulose and Lignin Content

**Figure 11:**
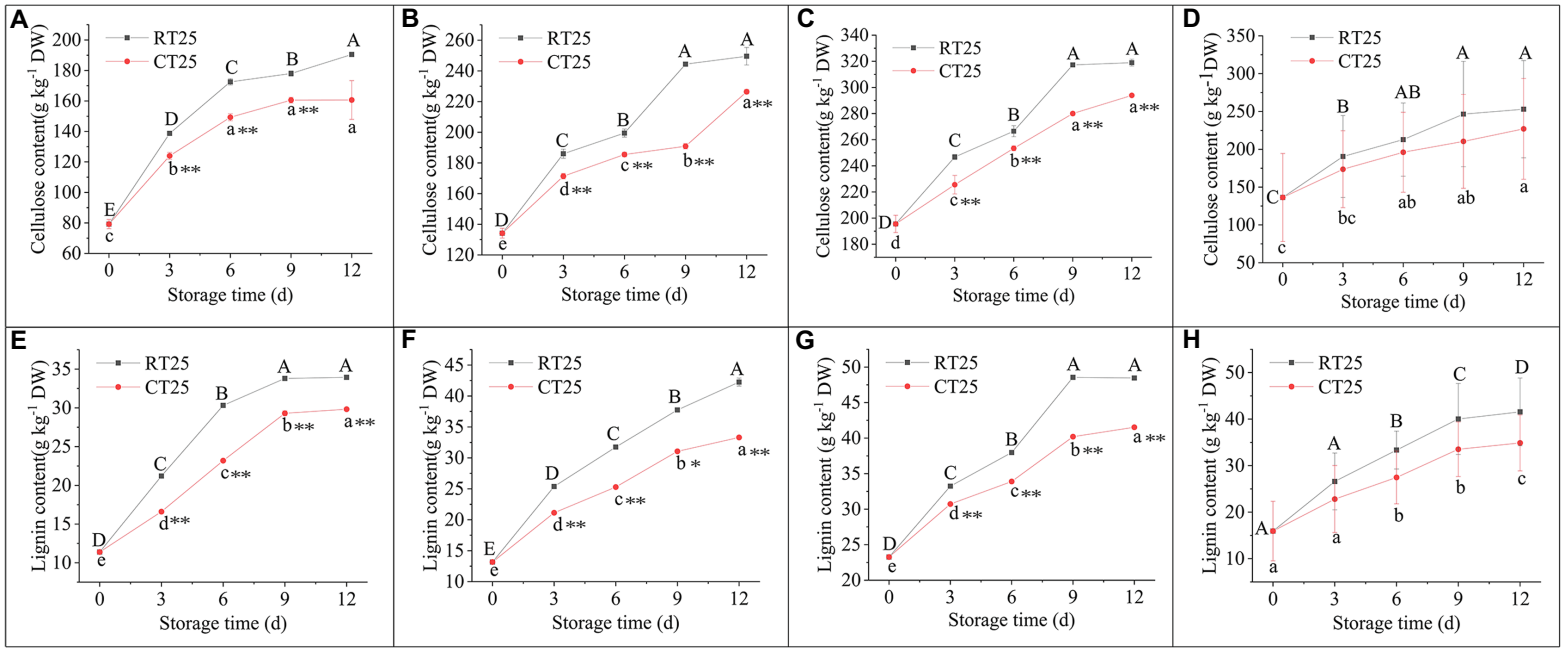
Dynamic changes in cellulose and lignin content in shoots during storage at 25°C and 4°C. RT25, bamboo shoots stored at room temperature and analyzed at 25°C; CT25, bamboo shoots stored at cold temperature and analyzed at 25°C. **(A–D)** Cellulose content in shoots during storage for upper **(A)**, middle **(B)**, and bottom **(C)** plant parts, and mean values **(D). (E–H)** Changes in lignin content in shoots during storage for upper **(E)**, middle **(F)**, and bottom **(G)** plant parts, and mean values **(H)**. Mean values were the mean values of upper, middle, and bottom plant parts from different treatments. Data are presented as mean±standard deviation (*n*=3). Significant analysis was performed using the Student’s *t*-test, in which different letters represent significant difference between treatments within the group, and the asterisk represents the level of significance between groups (^*^means the difference is significant at the 0.05 level, ^**^means the difference is significant at the 0.01 level).

During the entire storage period, lignin and cellulose content increased consistently, whether stored at 25°C or at 4°C (Figure 11). On a dry-weight (dw) basis, the cellulose content in bamboo shoots stored at RT increased significantly from 7.92 to 19.05% dw in the upper parts, from 13.42 to 24.96% dw in the middle parts, and from 19.56 to 31.90% dw in the bottom parts during the entire storage period (Figures 11A–D). For shoots stored under CT conditions, the cellulose content increased significantly to 16.06, 22.64, and 29.39% dw, respectively, in the upper, middle, and bottom parts, which is less than for those stored under RT conditions.

The lignin content of the shoots stored under RT conditions increased significantly from 1.14 to 3.39% dw in the upper, from 1.32 to 4.23% dw in the middle, and from 2.33 to 4.85% dw in the bottom after the 12d of storage (Figures 11E–H). Under CT conditions, the lignin content reached 2.98, 3.33, and 4.15% dw, respectively, in the upper, middle, and bottom shoot parts, which is less than that of those stored in RT conditions.

Although low temperatures can attenuate the accumulation of cellulose and lignin in the shoots, their content still increases consistently with storage time. The content of cellulose and lignin increased 1.86-fold and 2.62-fold, respectively, under RT during the entire storage period, but increased by 1.67-fold and 2.19-fold, respectively, under CT. In addition, both cellulose and lignins accumulated more rapidly during the first 3d of storage. For the shoots stored under CT conditions, more cellulose and lignins accumulated in the bottom parts, and the upper parts were still edible. However, bamboo shoots stored under RT conditions were completely brown and lost their edible value.

### Correlation Analysis Between Different Physiological Indexes During Storage

**Figure 12:**
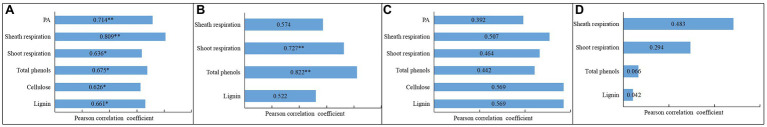
Pearson correlation analysis between different physiological factors during shoot browning and the aging process. ^*^ indicates a significant correlation, and ^**^ indicates an extremely significant respiration **(A)** Pearson correlation for NSC degradation between the dynamics of PA, cellulose, and lignin content and the dynamics of shoot and shoot respiration rates during storage at 25°C **(B)** Pearson correlation between PA content and the dynamics of lignin and total phenol contents and the dynamics of shoot and sheath respiration rates during storage at 25°C **(C)** Pearson correlation for NSC degradation between the dynamics of PA, cellulose, and lignin content and the dynamics of shoot and shoot respiration rates during storage at 4°C **(D)** Pearson correlation for PA content between the dynamics of lignin and total phenol content and the dynamics of shoot and sheath respiration rates during storage at 4°C.

By comparison, we noticed that the dynamic changes of NSC and PA content in shoots coincided well with those of respiration rates and total phenol, cellulose, and lignin content during storage. Hence, a Pearson correlation analysis was performed to analyze the relationships between the degradation of NSC and PA and the dynamics of other physiological indexes in bamboo shoots during storage (Figure 12).

Correlation analysis showed that the degradation of NSC correlated significantly with an increase in PA content and sheath respiration rates. Significant correlations were also shown for increases in shoot respiration rates and total phenol, cellulose, and lignin content under RT storage conditions (Figure 12A). The dynamic changes in PA content in bamboo shoots also correlated significantly with the dynamics of shoot respiration rates and total phenol content, but did not correlate significantly with lignin content during storage (Figure 12B). Under CT conditions, the NSC content showed significant correlation with the content of cellulose and lignin during storage (Figure 12C), but the PA content was not significantly correlated with the content of total phenols and lignins or the respiration rates of shoots in storage, implying that the degradation of PA was inhibited by the low temperatures (Figure 12D).

**Figure 13:**
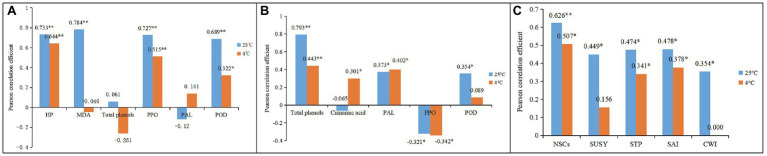
Pearson correlation analysis of the physiological indexes between different storage conditions. ^*^ Indicates a significant correlation, and ^**^ indicates extremely significant respiration **(A)** Pearson correlation among the substances of browning **(B)** Pearson correlation among the substances of lignification. **(C)** Pearson correlation among the substances of fibrosis.

In addition, browning rates were significantly and positively correlated with H_2_O_2_ and MDA content and with PPO and POD activity but were not significantly correlated with total phenol content or PAL activity in the shoots during storage at 25°C (Figure 13A). A similar trend was found for shoots stored at 4°C, but the correlation coefficients decreased. This might be due to low temperatures inhibiting the production of H_2_O_2_ and MDA and the activity of browning-related enzymes in bamboo shoots, which further attenuated their browning rates.

The dynamic changes in lignin content were also significantly and positively correlated with the dynamic changes in total phenol content and PAL and POD activity but had significant negative correlations with PPO activity in shoots during storage at 25°C (Figure 13B). For shoots stored at 4°C, the lignin content was significantly correlated with cinnamic acid content (Figure 13B), which was different than it was for those stored at 25°C. These results are probably due to more phenol flowing into the lignin-synthesis pathway, rather than into the browning reaction under CT conditions. The significantly decreased browning rates in bamboo shoots stored at 4°C support this deduction.

The cellulose content in the shoots during storage correlated significantly with NSC content and with the activity of SUSY, STP, SAI, and CWI at 25°C (Figure 13C). However, for shoots stored at 4°C, cellulose content no longer correlated significantly with the activity of SUSY and CWI. SUSY catalyzes sucrose and uridine diphosphate (UDP) into UDP glucose, which is a soluble precursor for the synthesis of cellulose and hemicelluloses (Moscatello et al., 2011; Wang et al., 2020). Therefore, low temperatures can attenuate the synthesis of cellulose in bamboo shoots by inhibiting precursor production and related enzymatic activity during storage.

## Discussion

Fresh-cut products are more perishable than intact commodities during storage because of wound-induced physiological changes (Lu and Xu, 2004). As a traditional forest vegetable, with a long history of consumption, it is difficult to enhance the shelf life of bamboo shoots because of senescence and quality loss, including weight loss, nutrient degradation, browning, lignification, and fibrosis.

### Weight Loss, Respiration, and Nutrient Degradation During Storage

The bamboo shoots lost significant weight with storage time, and the percentage of fresh-weight loss increased consistently, which is most likely due to constant respiration, water loss, and degradation of NSCs and PAs during storage.

As a rapidly growing vegetative organ, bamboo shoots still showed strong physiological activity after harvest. When the fresh-cut shoots were sealed in plastic bags, the bags soon swelled and were full of air, which suggests a strong respiration reaction in the postharvest shoots. In addition, mechanical injury can cause significant physiological changes and increase the respiration rate in fresh-cut shoots (Lu and Xu, 2004). Yu et al. (2007) reported that the respiration rate of *Dendrocalamopsis oldhamii* shoots reached their maximum values in 4–6h after harvest and then decreased consistently. In the present study, the respiration rate was determined every 3d and consistently decreased. The postharvest *F. yunnanensis* shoots appeared to reach their maximum respiration rate during the first 3d and then declined thereafter, which requires further confirmation.

Respiration rate is an indicator of the potential shelf life of fresh-cut products (Church and Parsons, 1995) and is an enzyme-controlled process involved in the breakdown of nutrients, which release energy for metabolic activity (Okonkwo, 1998). The shoots in our study could still elongate slightly during storage. All biological activity in bamboo shoots consumes a great deal of energy, which is mainly supplied by the degradation of NSCs (starches and soluble sugars) and PAs (soluble proteins and amino acids). Therefore, the content of NSCs and PAs in shoots decreases throughout the entire postharvest storage period, especially during the first 3d, in which it decreases sharply.

Moreover, the activity of starch- and sucrose-metabolizing enzymes in shoots decreased significantly after 3d, which implies that shoot quality deteriorated significantly during the first 3d. However, the shoots stored at 4°C had higher starch, soluble sugar, soluble protein, and amino acid content than those stored at RT throughout the entire storage period. For shoots stored at CT, higher respiration rate and greater activity by the starch- and sucrose-metabolizing enzymes was shown when determined at RT but had much lower values when determined directly at CTs. These shoots also showed higher starch, soluble sugar, soluble protein, and amino acid content than was shown by those stored at RT. These results indicate the favorable effects of low temperature at keeping shoots fresh during postharvest storage by significantly inhibiting the related enzymatic activity, which recovered quickly at RT.

### Browning During Storage

Under RT conditions, fresh-cut shoots turned brown rapidly in the middle and bottom plant parts within 3–6d and then completely browned after 6–9d. However, low-temperature storage conditions significantly inhibited the browning of shoots during storage, and shoots had a better appearance at the end of storage. Accordingly, browning-related physiological indexes, such as H_2_O_2_, MDA, total phenol, and related enzymatic activity, also had lower values at the end of storage.

Browning of harvest crops correlates with senescence, water loss, chilling injury, mechanical damage, and other stresses and usually increases the production of reactive oxygen species (ROS; Neill et al., 2002; Chen et al., 2013; Lin et al., 2002, 2016). Respiration is also a major source of ROS, which is mainly generated in the mitochondria (Purvis et al., 1995; Mittler, 2002). As a species of ROS, H_2_O_2_ stimulates membrane lipid peroxidation, disrupts cellular membrane structures, results in PPO and POD contacting the phenolic substrates, and oxidizes phenolics to form brown polymers (Lin et al., 2005; Wu et al., 2006; Chen et al., 2013). MDA is usually employed to analyze the degree of membrane lipid peroxidation (Lin et al., 2005). In this study, the shoots stored at 25°C showed increases in MDA and H_2_O_2_ content, which is consistent with the browning changes of the shoots. Our analyses also showed significant positive correlation between browning rates and H_2_O_2_ and MDA content during the postharvest storage of bamboo shoots. These results imply that the increasing MDA and H_2_O_2_ content stimulated the browning of bamboo shoots during storage. Similar results were obtained with lychee (*Litchi chinensis*) during storage, who considered that the pericarp browning of harvested lychee fruit was associated with a rapid increase in the content of H_2_O_2_ and hydroxyl radicals (Ruenroengklin et al., 2009; Bhushan et al., 2015; Zhang et al., 2018; Yun et al., 2021). H_2_O_2_ dramatically stimulates membrane lipid peroxidation, increases the accumulation of ROS, and accelerates the browning of the pericarp of longan (*Dimocarpus longan*; Lin et al., 2016, 2017, 2020; Wang et al., 2018c). We conclude that the accumulation of H_2_O_2_, together with MDA, can significantly accelerate the browning rates of bamboo shoots during storage.

The browning of bamboo shoots is enzymatic (Zhang et al., 2000), and oxygen, the substrate involved, and the related enzymes are all essential to enzymatic browning (Shen et al., 2006; Luo et al., 2012a). In our study, total phenols increased in the beginning and then decreased during the last storage period, which was different from the trend for MDA and H_2_O_2_ content, probably because most phenols were oxidized into brown polymers or consumed to synthesize lignins. In addition, the death of shoot tissues caused by the browning might reduce the synthesis of total phenols. Martinez and Whitaker (1995) considered enzymatic browning at injury sites in fruit and vegetables to be an activity that could be managed by controlling PAL activity and thereby the biosynthesis of phenolic compounds. However, the present study showed no significant positive correlation between total phenols and the browning of bamboo shoots during storage. Our study showed PAL activity and browning results to be similar, which is different from the previous study on vegetables and fruits (Martinez and Whitaker, 1995), perhaps, because significant lignification of bamboo shoots consumed more phenolic compounds during storage than occurred for other fruit and vegetables. Phenols are not only the substrate for enzymatic browning but also the substrate for lignin formation. Boerjan et al. (2003) found that PAL catalysis is the first reaction in the phenylpropanoid biosynthesis pathway and the first enzyme involved in lignin cell-wall deposition. The formation of lignins caused lower correlation coefficients between the rate of bamboo shoot browning, total phenol content, and PAL activity as compared with other physiological indexes, such as MDA and H_2_O_2_ content and PPO and POD activity during storage. Therefore, these results do not contradict the previous findings that the most important factors determining the browning of fruit and vegetables are the concentrations of active PPO and phenolic compounds (Martinez and Whitaker, 1995). In fact, lignification seldom occurs in fruit, so most of its phenols are converted into browning compounds, increasing the significant correlation between PPO activities and browning reactions, which is supported by the fact that bamboo shoots stored at low temperature could still lignify, whereas browning was almost completely inhibited for a dozen days.

Browning in fruit and vegetables is initiated by the enzymatic oxidation of phenolic compounds by PPO (Martinez and Whitaker, 1995; Wesche-Ebeling and Montgomery, 2010; Moon et al., 2020; Yang et al., 2020). Tian et al. (2002) reported that controlled atmospheric conditions can significantly inhibit PPO activity and effectively prevent browning in longan fruit. POD participates in the oxidation and accumulation of phenolic materials, resulting in browning (Wang, 1990). Shen et al. (2006) reported that the browning of bamboo shoots could be increased by increasing the POD activity and could be prevented by inhibiting its activity. In the present study, PPO and POD activities showed significantly positive correlation with the browning rate of bamboo shoots during their postharvest storage, which supports the earlier findings by Mayer (2006) that the browning in plants induced by mechanical damage is attributable to PPO activity, but differs from the results of Aquino-Bolaños et al. (2000) and Aquino-Bolaños and Silva (2004), who found that PPO was not directly involved in the browning process in cut jicama (*Pachyrhizus erosus*).

Browning is an important cause of impaired quality during the postharvest storage of fruit and vegetables (Luo et al., 2007; Fan et al., 2016; Moon et al., 2020). Some of the polyphenolic compounds are substrates for PPO (Deutch, 2018). PPO oxidizes polyphenols to quinones, which form melanin and cause pulp to brown (Toivonen and Brummell, 2008). The activities of PPO and the concentrations of polyphenolic substrates are related to the degree of browning (Holderbaum et al., 2010). However, total phenols increased in the beginning and then decreased during the last storage period, and the present study showed no significant positive correlation between total phenols and the browning of bamboo shoots during storage, probably because most phenols were oxidized into brown polymers or consumed to synthesize lignins. When stored at 4°C, the browning of bamboo shoots was completely prevented during the entire storage period. Cold storage condition inhibited the increase of MDA, H_2_O_2_, and total phenol contents, and reduced the activities of PAL, POD, and PPO, which lessened shoot browning. The low temperature might inhibit membrane-lipid peroxidation of bamboo shoots, better maintaining cell-membrane integrity during storage by inhibiting the production of H_2_O_2_ and alleviating the disruption and compartmentalization of cellular membranes by decreasing the contact between enzymes and substrates. Lower correlation coefficients between browning rates and MDA content in bamboo shoots stored at 4°C support this result. Low temperatures decrease the activity of PAL, PPO, and POD and inhibit the increase of phenols and other browning compounds in postharvest shoots during storage.

### Fibrosis and Lignification During Storage

Bamboo shoots are not easily stored because they tend to lignify in addition to browning (Shen et al., 2006). Li et al. (2019b) considered lignification a typical senescence characteristic in bamboo shoots during storage. Accumulated evidence demonstrates that PAL and POD have fundamental roles in lignification (Yang et al., 2010; Luo et al., 2012b; Wang et al., 2019). The lignification of plants involves the polymerization of monolignols primarily derived from the phenylpropanoid pathway. That polymerization begins with the PAL reactions that form cinnamate derivatives and ends with the POD reactions that form the lignin polymer (Imberty et al., 1985; Boerjan et al., 2003). Upon perturbation of the phenylpropanoid pathway, pathway intermediates may successfully incorporate into the lignin polymer (Liu et al., 2018; Vanholme et al., 2019). The lignification of bamboo shoots showed significant positive correlations with the content of cinnamic acid and total phenols and PAL and POD activity, but negative correlations with PPO activity during storage. This is significantly different from the results for browning rates, which showed negative or low correlation with PAL activity but significant positive correlation with PPO activity. Luo et al. (2008, 2012b) also reported significant positive correlation between lignin content and PAL and POD activities. Because of the lignin formation in postharvest bamboo shoots, total phenol content decreased in the last storage period under RT conditions. As a result, the correlation coefficient between the browning rate, total phenol content, and related enzyme activity decreased.

Generally, there is a close relationship between browning and lignification in postharvest shoots during storage. Aquino-Bolaños et al. (2000) found that browning on the surface of jicama was caused by the lignification process or by wound healing in damaged areas. As the initial substrate for lignin formation, cinnamic acid showed inapparent correlation with lignin content in bamboo shoots under RT but showed significant correlation under CT. This result further supports our inference that phenols were involved in the browning reaction under RT, but their correlation coefficient with lignin decreased during storage. During CT, the browning reaction in bamboo shoots was inhibited, and the cinnamic acids mainly participated in the lignin-formation pathway; therefore, significant correlation was seen between cinnamic acid and lignin. There is a competitive relationship in phenol consumption between the browning reaction and lignification in bamboo shoots during storage.

Taste evaluation is an effective way to judge what consumers like or dislike, and chemical components of the cell walls, such as cellulose, hemicellulose, and lignin, have a central role in determining textural quality (Wang et al., 2018b). Wang (2002) reported cellulose was largely synthesized during the postharvest storage of tortoise-shell bamboo (*Phyllostachys edulis*) shoots. Similarly, *F. yunnanensis* shoots show a consistent increase in cellulose contents during storage, which correlates positively with a decrease in NSC content and with the enzymatic activity of STP, SAI, CWI, and SUSY. STP is dominant in starch degradation during shoot development (Wang et al., 2019), catalyzing starch into glucose-1-phosphate, which is converted into glucose-6-phosphate or UDP glucose and consumed to synthesize fatty acids and cellulose or enters other metabolic pathways (Patrick et al., 2013; Bahaji et al., 2014). SUSY-catalyzed metabolism is related to the biosynthesis of the cell wall and starch (Winter and Huber, 2000). As a SUSY-catalyzed product, UDP glucose usually synthesizes cell-wall components, such as cellulose and hemicelluloses (Moscatello et al., 2011). SAI produces the monomeric sugars, in an early preparation for further internode elongation, and supplies monomeric sugars for fiber development. SAI and SUSY share in carbon metabolism (Moscatello et al., 2011; Wang et al., 2019). Therefore, the increase in cellulose content in postharvest bamboo shoots is significantly and positively correlated with the hydrolysis of NSCs, which are catabolized by STP, SUSY, SAI, and CWI. In addition, the activity of CWI is less than that of other enzymes and is completely inhibited during CT, which implies that CWI does not have an important role in NSC hydrolysis during storage. Low temperatures significantly decrease the activity of STP, SUSY, SAI, and CWI in bamboo shoots during storage and further mitigate the increase in cellulose content, reducing the correlation between cellulose content and the related catabolizing enzymes. By comparison, the inhibitory effects of low temperature on browning in bamboo shoots are more significant. Interestingly, we found that the enzyme activity recovered rapidly from low temperature to room temperature. Especially, in the later stage of cold storage, the activity of some enzymes, such as sucrose- and starch-catabolizing enzymes and PAL, was even higher than that of storage at 25°C. Therefore, we recommend that bamboo shoots should be placed on refrigerated shelves, and once they were removed from cold conditions, the chilled bamboo shoots should be consumed rapidly because enzyme activity recovered quickly.

Similar to other physiological indexes, the content of both lignins and cellulose increased sharply during the first 3-d period, indicating that postharvest shoots should be sold within 3d under RT conditions; otherwise, the mouthfeel and appearance deteriorate quickly. However, shoots stored at 4°C have a significantly extended shelf life of 6d, according to their accumulated levels of lignin and cellulose and their browning rates, as well as their tastiness. Postharvest shoots, whether stored at 25°C or at 4°C showed a consistent increase in cellulose and lignin content throughout the entire storage period, which contradicts the conclusions reported by Lu and Xu (2004), who suggested that cellulose and lignin decomposed during storage. They considered the decomposition of cellulose and lignin as one of the reasons bamboo shoots softened during storage. Actually, cellulose and lignin do not easily biodegrade under natural conditions because of their stability and complex chemical structure (Boswell, 1941; Reid, 1995; Yang et al., 2007; Cao et al., 2018; Shrotri et al., 2018). Instead, the softening of bamboo shoots is caused by the destruction of the cell structure during storage.

### Correlation Analysis of the Metabolic Flow of NSCs and PAs During Storage

**Figure 14:**
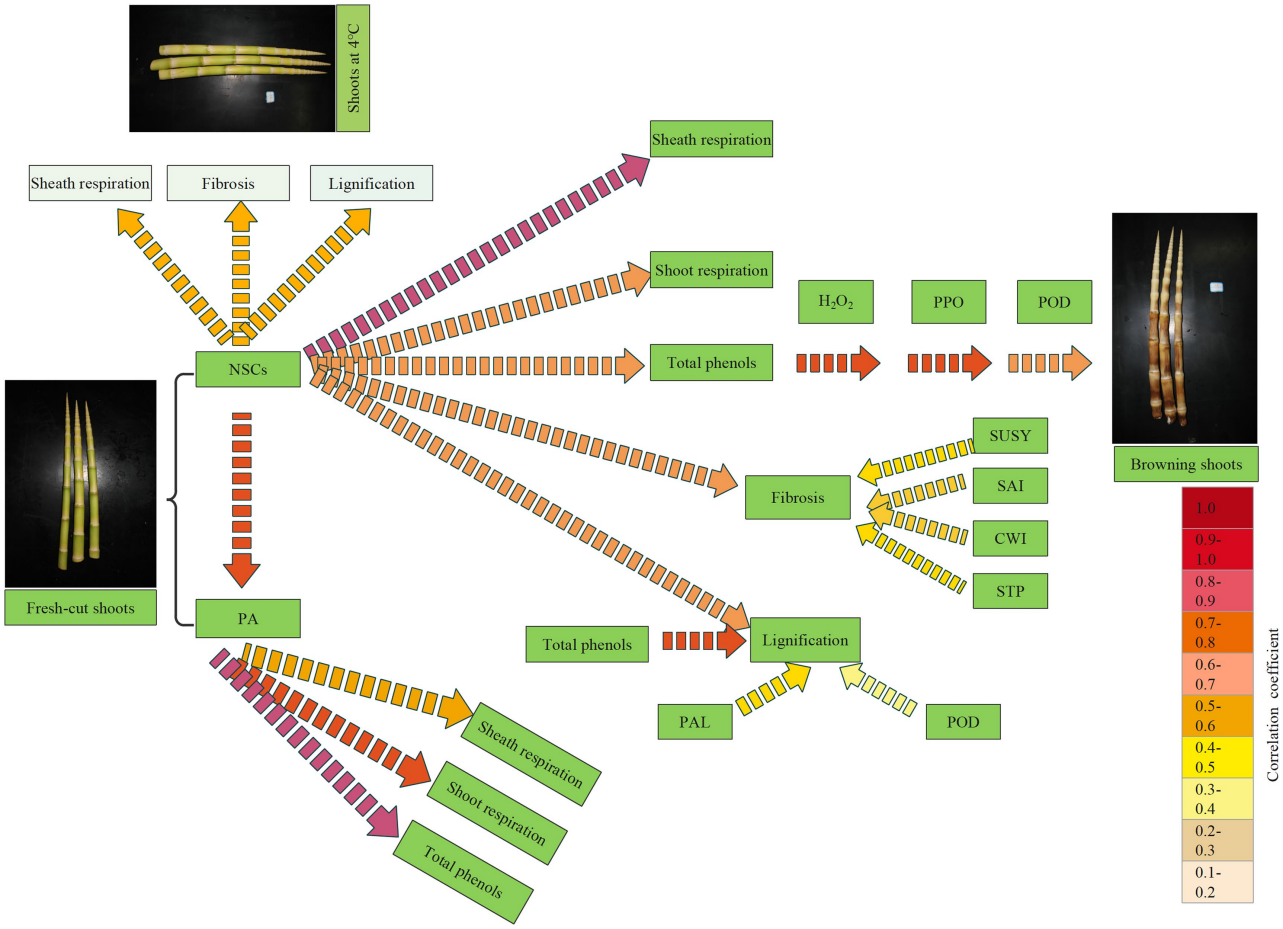
A metabolite flow diagram based on the correlation analysis among physiological indices of postharvest shoots during storage. Darker colors for the dotted arrows shown increasing correlation.

The carbohydrates, soluble proteins, and amino acids in bamboo shoots degrade constantly and are consumed during life activities, such as respiration, browning, substance transformation, energy consumption, and fibrosis and lignification. To analyze the nutrient consumption and exhibit the probable metabolic flow in bamboo shoots during storage, we established a metabolite flow diagram based on correlation analysis among physiological indexes determined in the present study (Figure 14). Significant correlation reflects the metabolic flow of NSCs or PAs in shoots during storage. The higher the correlation coefficients are, the greater is the probability of metabolite flow in that pathway.

During the storage period at 25°C, the NSCs of bamboo shoots degrade and mainly flow into the synthesis of total phenols, lignins, and cellulose *via* the glycolysis, pentose phosphate, and shikimic acid pathways (Wang and Cui, 2009). The NSCs might also be consumed through the respiration of shoot sheaths and shoots based on the high and significant correlation coefficient with that process. The shikimic acid pathway involves the synthesis of phenolic compounds in plants, converting simple carbohydrate precursors derived from glycolysis and the pentose phosphate pathway into the aromatic amino acids phenylalanine and tryptophan (Mandal et al., 2010). Phenols form brown polymers under the oxidization and catalysis of H_2_O_2_, PPO, and POD, resulting in the browning of bamboo shoots during storage. NSCs were catabolized by STP, SUSY, SAI, and CWI; and then, cellulose was synthesized, resulting in fibrosis of the bamboo shoots. Meanwhile, phenols are also involved in lignin synthesis through the catalysis of PAL and POD. The content of PAs in postharvest shoots decreases consistently with storage time and is mainly consumed *via* the respiration of shoot sheaths and shoots by having a role in the synthesis of the phenols. Previous study has shown that shoot sheaths are an important respiration organ in bamboo shoots (Wang et al., 2018a).

Under CT storage conditions, NSCs in bamboo shoots mainly flow into sheath respiration, fibrosis, and lignification, as shown by their significant correlation coefficients (Figure 13). Meanwhile, the formation of phenols in the bamboo shoots and their browning are inhibited, and the degradation of PA is also mitigated. Hence, total phenols do not show significant correlation with NSC and PA content in bamboo shoots under CT storage conditions. Low temperatures can better maintain fresh-cut shoots by inhibiting the degradation of NSCs and PAs during storage. Therefore, cold-chain transportation has been widely adopted to extend the shelf life of fresh-cut fruit and vegetable products, reducing respiration, nutrient degradation, and browning. However, once bamboo shoots are removed from CT conditions, the senescent- and browning-related enzymes rapidly recover, and the stored bamboo shoots should be sold or eaten quickly.

## Conclusion

The quality of *F. yunnanensis* shoots deteriorates rapidly in the first 3d at RT, which includes significant weight loss, browning, NSC and PA degradation, lignification, and fibrosis. Storage at 4°C increases shoot preservation. Under RT storage conditions, NSCs in shoots are consumed and mainly flow into sheath and shoot respiration, browning-related substances, fibrosis, and lignification, and PAs are mainly converted into the substances of respiration and phenols, whereas total phenols can be oxidized into brown polymers or consumed to synthesize lignins (Figure 14). Cold storage effectively attenuates the weight loss, respiration rates, and nutrient catabolism; inhibits the synthesis of phenols and the browning; and mitigates the lignification and fibrosis, delaying the aging and deteriorating processes. Cold storage effectively preserves fresh-cut bamboo shoots, better maintaining their appearance and nutrients for 6d.

## Data Availability Statement

The original contributions presented in the study are included in the article/supplementary materials, further inquiries can be directed to the corresponding author.

## Author Contributions

LY analyzed the data and participated in interpretation, and took part in writing the manuscript. JP performed most of the experiments, analyzed the data, and participated in interpretation. YZ collected and processed the samples, analyzed the data, and participated in interpretation. SW designed the project, provided supervision, and took part in writing the manuscript. All authors have read and approved the final version of the manuscript.

## Conflict of Interest

The authors declare that the research was conducted in the absence of any commercial or financial relationships that could be construed as a potential conflict of interest.

## Publisher’s Note

All claims expressed in this article are solely those of the authors and do not necessarily represent those of their affiliated organizations, or those of the publisher, the editors and the reviewers. Any product that may be evaluated in this article, or claim that may be made by its manufacturer, is not guaranteed or endorsed by the publisher.
